# Inhibition of indoleamine 2,3-dioxygenase-mediated tryptophan catabolism accelerates collagen-induced arthritis in mice

**DOI:** 10.1186/ar2205

**Published:** 2007-05-18

**Authors:** Sándor Szántó, Tamás Koreny, Katalin Mikecz, Tibor T Glant, Zoltán Szekanecz, John Varga

**Affiliations:** 1 Section of Molecular Medicine, Department of Orthopedic Surgery, Rush University Medical Center, Cohn Research Building, Room 708, 1735 W. Harrison, Chicago, IL 60612, USA; 2 Institute of Medicine, Division of Rheumatology, University of Debrecen, Medical and Health Science Center, 22 Móricz Street, Debrecen, H-4012, Hungary; 3 Division of Rheumatology, Northwestern University Medical School, 303 East Chicago Ave., Chicago, IL 60611, USA

## Abstract

Indoleamine 2,3-dioxygenase (IDO) is one of the initial and rate-limiting enzymes involved in the catabolism of the essential amino acid tryptophan. In cultured cells, the induction of IDO leads to depletion of tryptophan and tryptophan starvation. Recent studies suggest that modulation of tryptophan concentration via IDO plays a fundamental role in innate immune responses. Induction of IDO by interferon-γ in macrophages and dendritic cells results in tryptophan depletion and suppresses the immune-mediated activation of fibroblasts and T, B, and natural killer cells. To assess the role of IDO in collagen-induced arthritis (CIA), a model of rheumatoid arthritis characterized by a primarily Th1-like immune response, activity of IDO was inhibited by 1-methyl-tryptophan (1-MT) *in vivo*. The results showed significantly increased incidence and severity of CIA in mice treated with 1-MT. Activity of IDO, as determined by measuring the levels of kynurenine/tryptophan ratio in the sera, was increased in the acute phase of arthritis and was higher in collagen-immunized mice that did not develop arthritis. Treatment with 1-MT resulted in an enhanced cellular and humoral immune response and a more dominant polarization to Th1 in mice with arthritis compared with vehicle-treated arthritic mice. The results demonstrated that development of CIA was associated with increased IDO activity and enhanced tryptophan catabolism in mice. Blocking IDO with 1-MT aggravated the severity of arthritis and enhanced the immune responses. These findings suggest that IDO may play an important and novel role in the negative feedback of CIA and possibly in the pathogenesis of rheumatoid arthritis.

## Introduction

Locally produced proinflammatory cytokines such as tumor necrosis factor-α, interleukin (IL)-1, and IL-6 play a pivotal role in the pathology of rheumatoid arthritis (RA). These cytokines, by upregulation of several genes, are responsible both for the recruitment and continuous activation of the inflammatory cells and for inducing production of the enzymes that destroy bone and cartilage. However, these inflammatory events need to be balanced by the production of endogenous inhibitors, as inflammatory responses are generally localized and the consequent destruction of affected joints is less severe [1].

The ubiquitously expressed heme enzyme indoleamine 2,3-dioxygenase (IDO) catalyzes the non-hepatic oxidative degradation of tryptophan, the initial and rate-limiting step in tryptophan metabolism, resulting in depletion of this least abundant essential amino acid. Tryptophan starvation enables the host to restrict the growth of intracellular pathogens [2]. The expression of IDO in healthy tissues is generally quite low but is markedly upregulated in response to infection and inflammation. Recent studies have established an entirely novel important biological function for IDO. These studies indicate that IDO-mediated tryptophan depletion *in vitro *results in inhibition of matrix-degrading metalloproteinase enzyme production, suppression of inflammatory responses, and promotion of immune tolerance [3,4]. Accordingly, an emerging immunological paradigm regards IDO as a key regulatory control enzyme in both innate and adaptive immune responses. Indeed, modulation of cellular IDO expression and/or activity is now increasingly implicated in inflammation and autoimmunity, organ transplantation and fetal rejection, and evasion of immune surveillance by tumor cells. Furthermore, agents that inhibit IDO are under active investigation as potential adjuvants for tumor immunotherapy [5].

IDO-mediated tryptophan catabolism results in depletion of tryptophan with concomitant generation of kynurenine and ultimately NAD (nicotinamide adenine dinucleotide). Expression of the IDO gene is induced in most cell types in response to infection with microbial agents via activation of toll-like receptors. Furthermore, interferon-gamma (IFN-γ), IL-10, and cytotoxic T lymphocyte-associated antigen-4 (CTLA-4) have also been shown to stimulate IDO [4,6]. In contrast, IL-4, IL-13, and transforming growth factor-β are suppressors of IDO [7]. In previous studies, we have shown that IFN-γ caused time-dependent induction of IDO gene expression and activity in cultured normal fibroblasts [4]. The consequent tryptophan catabolism and ensuing tryptophan starvation in these cultures were associated with profound suppression of collagenase and stromelysin gene expression induced by IL-1β. Abrogation of IL-1β-induced matrix metalloproteinase stimulation upon activation of IDO in these fibroblasts was directly due to the reduction of local tryptophan concentrations rather than the accumulation of kynurenine and other tryptophan metabolites. These observations led us to propose the hypothesis that, through its ability to induce transient tryptophan starvation, IDO represented an important endogenous anti-inflammatory mechanism and that disruption of IDO induction or function would be associated with exaggerated inflammatory responses.

Collagen-induced arthritis (CIA) is generated in genetically susceptible mouse or rat strains by immunization with type II collagen (CII) dissolved in complete Freund's adjuvant (CFA). CIA highly resembles RA according to overlapping immunopathogenic pathways and similar histopathologic features [8]. Among other reasons, the central role of class II major histocompatibility complex [9], the involvement of CD4^+ ^T cells in the immunopathogenesis of the disease, the predominantly Th1 type immune response to CII in the induction phase [10,11], as well as the cytokine profile throughout the evolution of CIA [12] make this experimental animal model one of the most adequate models of RA. To address the question of whether IDO plays a negative regulatory role in an animal model of arthritis, we assessed IDO activity in the sera of mice at different stages of arthritis and the effect of 1-methyl-tryptophan (1-MT), a specific inhibitor of IDO, on arthritis development and antigen-specific immune responses in CIA.

## Materials and methods

### Immunization and assessment of collagen-induced arthritis in mice receiving either 1-methyl-tryptophan or vehicle

Mice were housed and bred under standard conditions at the Comparative Research Center of Rush University (Chicago, IL, USA). The Institutional Animal Care and Use Committee approved all animal experiments. DBA/1 male mice, 6 to 8 weeks of age, were purchased from The Jackson Laboratory (Bar Harbor, ME, USA). Mice were immunized by a standard immunization protocol. Briefly, 100 μg of human CII was emulsified in CFA (Difco Laboratories Inc., now part of Becton Dickinson and Company, Franklin Lakes, NJ, USA) and injected into the proximal tail of mice. A second injection of the same dose and adjuvant was given intraperitoneally (i.p.) on day 21. Mice that did not develop arthritis within 3 weeks of the second antigen injection were boosted with a third injection administered in equally divided doses i.p. and into the proximal tail. All mice were sacrificed 8 weeks after the first injection. After the second immunization, all mice were examined for swelling and erythema of distal joints. Paws were considered to have arthritis when swelling and erythema were noted in at least two digits and/or other joints. The clinical severity of arthritis was graded on a scale of 0 to 4 for each paw, according to swelling and redness. Specifically, scoring was performed as follows: 0 = healthy; 1 = mild swelling and erythema; 2 = moderate swelling and erythema; 3 = more intense erythema, swelling, and redness affecting a greater proportion of the paw; 4 = severe erythema, swelling, and redness affecting the entire paw. A cumulative score ranging from 0 to 16, based on individual paw scores of 0 to 4, was assigned for each animal.

Tablets containing slow release of D, L-1-MT (Innovative Research of America, Sarasota, FL, USA) or vehicle were surgically inserted under the dorsal skin of mice on days 22 and 29 after the first immunization. The tablets released 10 mg/day 1-MT for a period of 7 to 10 days.

### Measurement of antibody production and T-cell response

Antibodies to the immunizing human and mouse (self) cartilage CII were determined by enzyme-linked immunosorbent assay (ELISA), as described elsewhere [13-15]. Briefly, Maxisorp 96-well plates (Nalge Nunc, Naperville, IL, USA) were coated with 0.1 μg of human or mouse cartilage-derived CII in 100 μl of coating buffer. Antibodies were determined in serial dilutions of sera (1:500 to 1:62,500) using peroxidase-conjugated goat anti-mouse immunoglobulins G, A, and M (IgGAM) secondary antibodies (Zymed Laboratories Inc., now part of Invitrogen Corporation, Carlsbad, CA, USA). Serum antibody levels were expressed in milligrams per liter using mouse IgGAM as control (Invitrogen Corporation).

Antigen-specific T-cell responses, including IL-2 production and T-cell proliferation, were measured in quadruplicate samples of spleen cells (3 × 10^5 ^cells per well) cultured in the presence of 100 μg of collagen protein per milliliter. IL-2 was measured in supernatants harvested on day 2 by the proliferation of the IL-2-dependent CTLL-2 cell line. Antigen-specific T-cell proliferation was assessed on day 5 by the incorporation of ^3 ^[H]thymidine. In both cases, the antigen-specific response was expressed as stimulation index, which is a ratio of incorporated ^3 ^[H]thymidine (counts per minute) in silver-stimulated cultures relative to counts per minute in non-stimulated cultures [13,14]. Antigen-specific IFN-γ and IL-4 production by T cells was determined in culture conditions identical to those described for T-cell proliferation in 4-day-old conditioned medium (2.5 × 10^6 ^mononuclear cells per milliliter) using capture ELISAs (R&D Systems, Inc., Minneapolis, MN, USA).

### High-performance liquid chromatography analysis

Serum tryptophan (T) and kynurenine (K) concentrations were measured simultaneously by high-performance liquid chromatography, as previously described [16]. As relatively high volumes of sera are needed for the determination of K and T, pooled sera were used, K and T concentrations were determined, and then K/T ratios were calculated.

### Statistical analysis

Analyses of the arthritis score and disease incidence at different time points were carried out using the non-parametric Mann-Whitney *U *test and chi-square contingency analysis, respectively. Student *t *test was used for statistical analysis of all other data. Analyses were performed using SPSS version 7.5 software package (SPSS Inc., Chicago, IL, USA). Significance was set at a *p *value of less than 0.05.

## Results

### Clinical and histological features of arthritis: increase of both the incidence and severity in mice treated with 1-methyl-tryptophan

**Figure 1:**
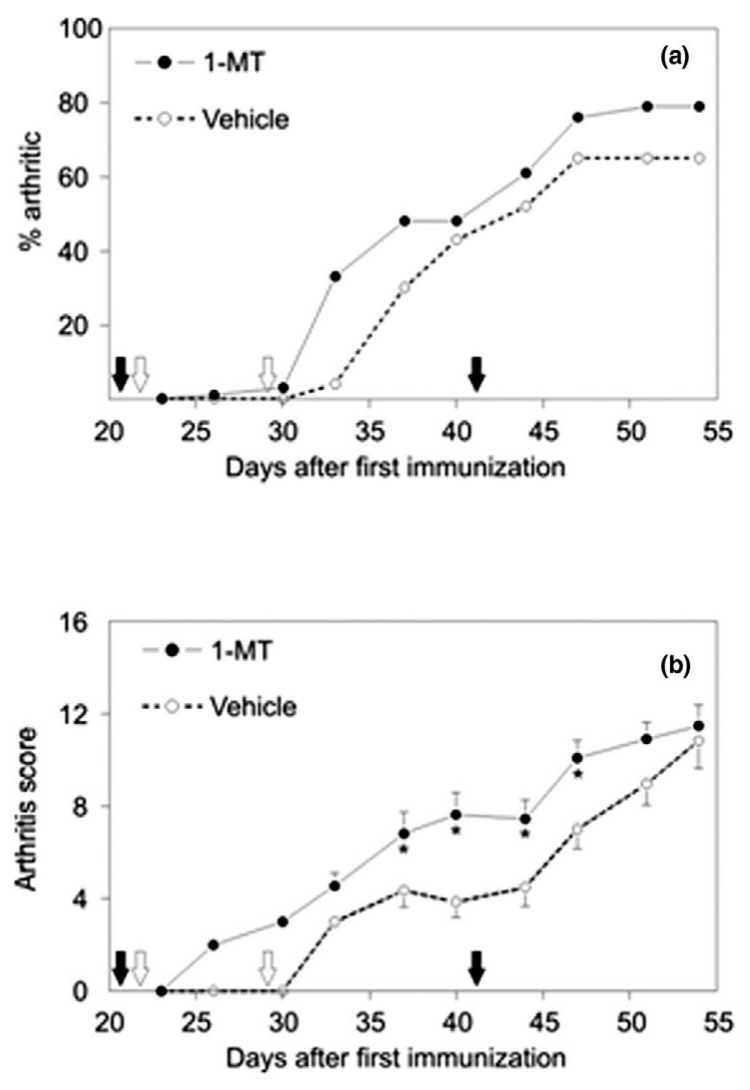
Incidence and severity of collagen-induced arthritis (CIA) in mice treated with 1-methyl-tryptophan (1-MT) or vehicle. Arthritis was first detected on day 26 after immunization with type II collagen on days 0, 21, and 42 (solid arrows) in mice treated with 1-MT or vehicle (open arrows). **(a) **Incidence of CIA expressed as the percentage of arthritic animals. **(b) **Disease severity expressed as the cumulative arthritis score in affected animals. Statistically significant differences in arthritis scores were found between days 37 and 47 (*p *< 0.05). Values are presented as the mean and standard error of the mean of 25 1-MT-treated and 20 vehicle-treated DBA/1 mice per group and represent two independent experiments.

**Figure 2:**
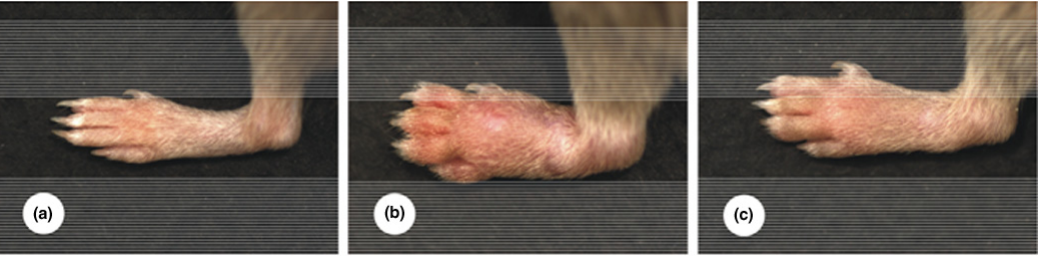
Hind paw images of DBA mice. **(a) **Hind paws of healthy non-immunized DBA mice. **(b) **Type II collagen-immunized (arthritic) DBA mice treated with 1-methyl-tryptophan 6 to 7 days after the onset of collagen-induced arthritis. **(c) **Type II collagen-immunized (arthritic) DBA mice treated with vehicle 6 to 7 days after the onset of collagen-induced arthritis. According to our scoring system (see Materials and methods), the three stages have been assigned scores of 0 **(a)**, 4 **(b)**, and 3 **(c)**.

Mice were immunized with CII and monitored for the development of arthritis for 54 days after the primary immunization. Clinical signs of arthritis typically appeared within 4 to 8 days after the second and third injections, which were administered on days 21 and 42 after the primary immunization, respectively. Treatment with 1-MT administered by the insertion of tablets containing 1-MT (or vehicle) on days 22 and 29 after the first injection resulted in an increased incidence of arthritis (Figure 1a). In addition to the higher disease incidence, the severity indicated by the cumulative arthritis score was significantly increased in mice receiving 1-MT as compared to those receiving vehicle between days 37 and 47 after the primary immunization (Figures 1b and 2). Histological analysis of the ankle, metatarsophalangeal, and interphalangeal joints of 1-MT-treated and vehicle-treated mice showed typical arthritis characterized by extensive leukocyte infiltration, synovial proliferation, pannus formation, and erosions. The severity of inflammatory cell infiltration and destruction of cartilage and bone reflected the clinical state, but no qualitative differences could be observed in mice receiving 1-MT compared to control mice.

### Cellular and humoral immune responses

**Figure 3:**
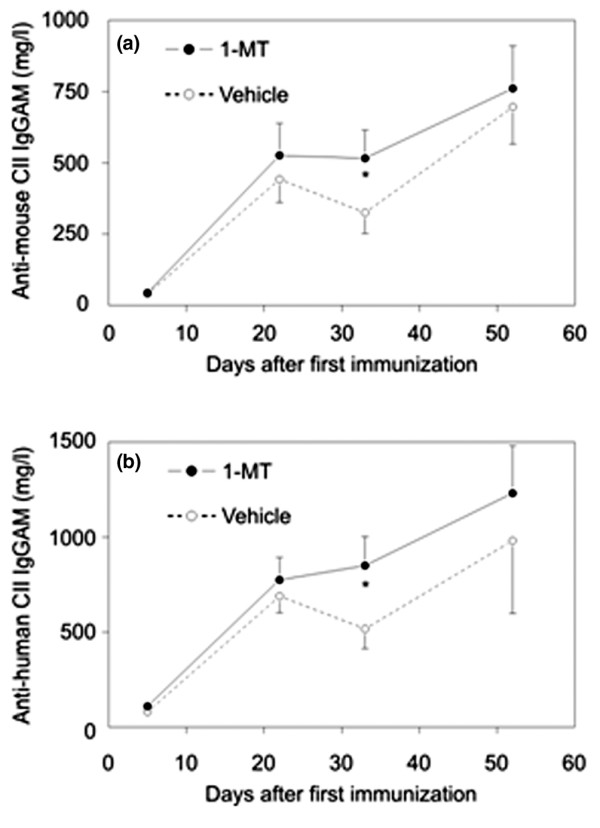
Humoral immune responses in DBA mice immunized with type II collagen (CII) and complete Freund's adjuvant (CFA) and treated with 1-methyl-tryptophan (1-MT) or vehicle. **(a) **Concentrations of antibodies (total concentrations of IgGAM [immunoglobulins G, A, and M]) to heterologous (human) CII were determined in the serum of DBA/1 mice immunized with CII and CFA and treated with 1-MT or vehicle. **(b) **Concentrations of antibodies (total concentrations of IgGAM) to autologous (mouse) CII were determined in the serum of DBA/1 mice immunized with CII and CFA and treated with 1-MT or vehicle. Sera were obtained on days 5, 22, 33, and 54. Values are presented as the mean and standard error of the mean of 25 1-MT-treated and 20 vehicle-treated DBA/1 mice per group and represent two independent experiments. *p < 0.05 between vehicle and 1-MT-treated mice on the corresponding days.

Because of the differences in incidence and severity of arthritis between mice receiving 1-MT or vehicle, it seemed to be prudent to detect immune responses to CII. Significant increases were found in both CII-specific autoantibody and heteroantibody titers in 1-MT-treated mice compared to the vehicle group; however, these differences disappeared by the end of the follow-up period (day 55) (Figure 3a,b). When assessing T-cell proliferation in the two groups at the end of follow-up, no differences in T-cell responses to heterologous CII, and only moderately increased T-cell proliferation to autologous CII in the 1-MT group, could be detected. Similarly to T-cell proliferation, IL-2 concentrations in the supernatants of spleen cells stimulated with mouse or human CII were slightly (but non-significantly) higher in the 1-MT-treated group than in vehicle-treated group at the end of the follow-up (data not shown).

### Spleen cell supernatant cytokine levels in mice with collagen-induced arthritis treated with 1-methyl-tryptophan or vehicle

**Figure 4:**
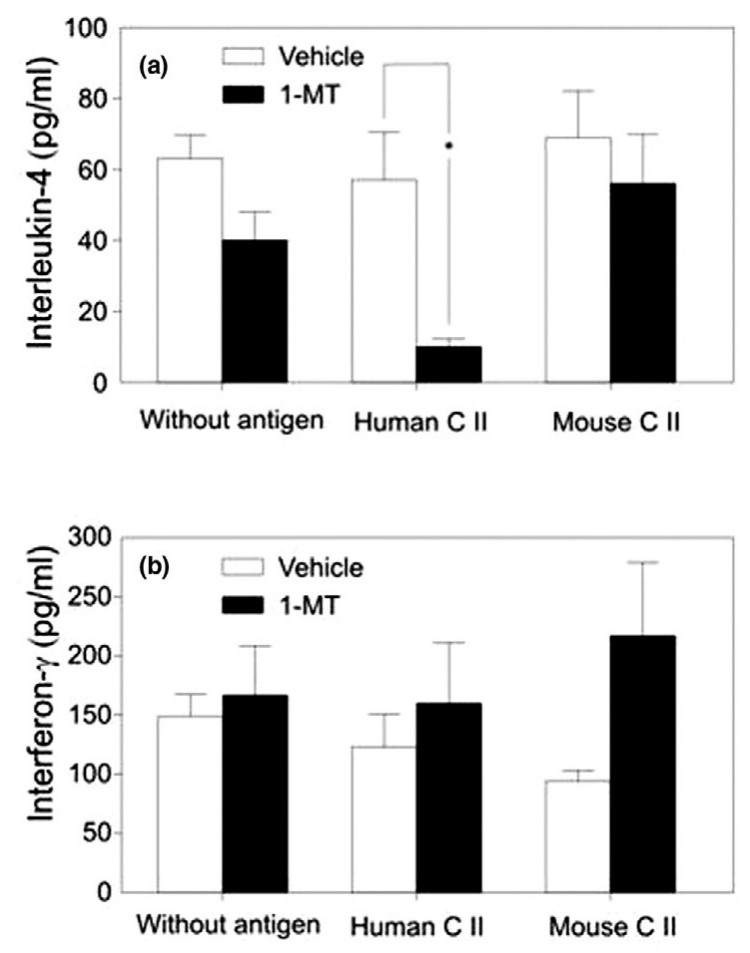
Concentrations of interleukin-4 (IL-4) and interferon-γ (IFN-γ) in the supernatants of spleen cells harvested from mice with collagen-induced arthritis and treated with 1-methyl-tryptophan (1-MT) or vehicle. The release of IL-4 **(a) **and IFN-γ **(b) **into the supernatants of spleen cells in response to human or mouse type II collagen (CII) was determined at the end of the experiment. Values are presented as the mean and standard error of the mean of 16 animals per group (*p < 0.01).

The concentrations of IFN-γ and IL-4 were determined in the supernatants of spleen cells from mice with CIA and treated with 1-MT or vehicle, respectively, at the end of the follow-up. Cytokine production of cells was assessed either without stimulation or after challenging with mouse or human CII. A lower concentration of IL-4 was detected in supernatants of spleen cells from mice treated with 1-MT in comparison to treated those with vehicle, but the difference was significant only when challenging the cells with human CII (*p *< 0.05) (Figure 4a). In contrast, slightly (but non-significantly) higher concentrations of IFN-γ could be measured in supernatants of spleen cells from 1-MT-treated mice compared to those from vehicle-treated animals (Figure 4b).

### Kynurenine/tryptophan ratios in collagen-induced arthritis mice treated with 1-methyl-tryptophan or vehicle

**Figure 5:**
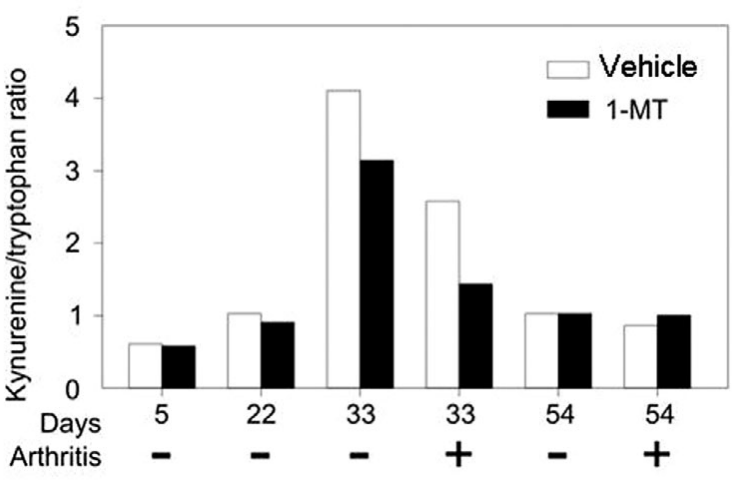
Kynurenine/trypophan (K/T) ratios in sera of DBA/1 mice immunized with type II collagen (CII) and complete Freund's adjuvant (CFA) and treated with 1-methyl-tryptophan (1-MT) or vehicle. K and T concentrations were determined and K/T ratios were calculated using pooled sera of mice immunized with CII and CFA. Each sample contained the sera of every mouse of the corresponding group in equal volume. Because most animals develop arthritis after day 30 (Figure 1), day 33 represents pre-symptomatic animals. As shown here at day 33, those animals, which subsequently will not develop arthritis (-), have much higher K/T ratio than those which will develop arthritis (+).

To estimate the enzymatic activity of IDO in different stages of arthritis, K and T concentrations were measured and K/T ratios were calculated in the pooled sera of mice treated with 1-MT or vehicle. Increased K and decreased T concentrations were measured, and thus increased K/T ratio was detected in mice with acute arthritis, but this ratio was even higher in immunized and immediate pre-arthritic mice (day 33). Thus, on day 33, animals that will not subsequently develop arthritis have higher K/T ratios in comparison to those that will develop arthritis. As was expected, treatment with 1-MT decreased the K/T ratio in the serum of arthritic and immediate pre-arthritic mice compared to the vehicle-treated ones. At the end of follow-up, the K/T ratio of mice was as low as that of the immunized mice. 1-MT treatment did not have any effect on K/T ratio (Figure 5).

## Discussion

RA and experimental inflammatory joint diseases have a progressive character with involvement of increasing numbers of joints; however, the initial and aggressive acute phase in affected joints slows down over time and the inflammatory processes burn out. Several lines of evidence indicate that the effector mechanism that initially attacks the joints is T cell-driven in response to the effect of proinflammatory cytokines, but the mechanisms responsible for the limitation of acute inflammatory processes are much less understood. The novel finding of our study is that IDO activity is upregulated in the acute phase of CIA reflected by the increased K/T ratio in the serum. Furthermore, we could also demonstrate that inhibition of IDO in this experimental model augments the incidence and severity of the disease and increases the immune responses to the autoantigens and alloantigens. These data suggest that IDO plays a central role in the negative regulatory feedback of immunological mechanisms in inflammatory joint diseases.

CIA, like RA in humans, is characterized by the accumulation of T cells, plasma cells, macrophages, B cells, mast cells, natural killer (NK) cells, and dendritic cells in the synovial sublining [17,18]. Furthermore, inflammatory cells infiltrating the synovial tissue in RA and in the acute phase of CIA exhibit a predominantly Th1 pattern of cytokine expression [10,11]. By priming the Th1-type inflammatory cell responses, IFN-γ is one of the most important proinflammatory factors in the induction of T cell-driven autoimmune arthritis, such as CIA. However, IFN-γ plays an ambiguous role in autoimmunity. After the activation of self-reactive lymphocyte clones and of bystander and accessory cells in the acute phase, IFN-γ downregulates the autoimmune processes [19]. Indeed, CIA and CFA developed more readily in IFN-γ receptor-deficient mice than in wild-type littermates [20]. As a possible explanation, it has emerged that CFA elicits strong myelopoiesis and expansion of Mac-1^+ ^cells, which play a crucial role in disease pathogenesis, and this process is downregulated by IFN-γ [20]. However, the exact mechanism responsible for the effect of IFN-γ in CIA has not been fully elucidated.

One of the most likely mechanisms for the downregulation of CIA by IFN-γ is the increased expression of IDO by non-T cells [21]. In fibroblasts [22], macrophages [23], and dendritic cells [3], IFN-γ stimulates the enzyme IDO, which degrades the amino acid tryptophan to form kynurenine, resulting in the inhibition of autoimmune processes. According to this assumption, we demonstrated an elevated K/T ratio, indicating high IDO activity during the acute phase of CIA. Moreover, the K/T ratio was even higher in mice that received CII and CFA but developed no clinical and histological signs of arthritis. These data suggest that IDO acts as a negative feedback in this model, and the onset and severity of experimental arthritis are inversely proportional to IDO activity.

To confirm the regulatory role of IDO in CIA, we used 1-MT, a known competitive inhibitor of IDO. 1-MT did not influence IFN-γ, but it significantly suppressed IL-4 production by spleen cells, resulting in an increased Th1/Th2 response. In 1-MT-treated mice, we could demonstrate a significant decrease of K/T ratio in the immediate pre-arthritic and acute phase of arthritis compared to vehicle-treated animals, suggesting the high activity of IDO only in these stages of inflammatory processes. In other words, the low blocking activity of 1-MT either in the pre-arthritic phase or in the chronic phase of arthritis denotes the anti-inflammatory effect of IDO only in the case of upregulation of inflammatory processes, especially Th1 responses.

As a tryptophan-catabolizing enzyme, IDO can induce the peripheral tolerance and reduce the persistent immune activation. On one hand, IDO decreases the tryptophan concentration in the microenvironment of inflammatory cells. Although tryptophan is an essential amino acid indispensable for the biosynthesis of proteins, the low tryptophan concentration results in the arrest of cell proliferation in the mid-G_1 _arrest point. T cells are specifically sensitive to tryptophan deprivation [2,5] and thus IDO activity can block the potential harmful autoimmune response. On the other hand, Zhu and colleagues [24] proposed that synovial T cells derived from RA synovial fluids are resistant to IDO-mediated tryptophan deprivation. This may be one mechanism by which autoreactive T cells are sustained *in vivo *in patients with arthritis [24]. In addition, selected metabolites on the tryptophan-kynurenine pathway are able to suppress proliferation of allogeneic T cells and, to a lesser extent, B and NK cells [25]. Moreover, some of the kynurenine derivates can induce *in vitro *the selective apoptosis of Th1 cells, but not Th2 cells [7]. In accordance with these results, 1-MT treatment in our study resulted in an increased, mainly Th1 cell-mediated immune response to the CII and consequently in the significant worsening of severity and increase of onset of CIA mediated by Th1 response.

In this regard, recent studies with the CIA model of arthritis using an antibody to the costimulatory molecule CD137, a member of the tumor necrosis factor receptor superfamily, are of great interest. These results showed that treatment of mice with the anti-CD137 antibody resulted in induction of IDO *in vivo*, which was associated with significant amelioration of the severity of CIA [26]. Furthermore, pharmacological inhibition of IDO reversed the effects of the anti-CD137 antibody and aggravated the arthritis in this model.

The tryptophan-IDO pathway may have important relevance for the biological therapy of RA. In a study of Boasso and colleagues [27], CTLA-4-Fc treatment of human peripheral blood CD4^+ ^T cells resulted in increased IDO expression by these cells. This effect was not observed in CD8^+ ^T cells. Thus, abatacept (CTLA4-Ig) therapy may act, at least in part, by the stimulation of IDO production.

## Conclusion

The importance of IDO activity in the regulation of CIA supports the hypothesis that IDO expression by antigen-presenting cells is responsible for suppression of undesirable Th1 cells in human inflammatory joint diseases, as tryptophan degradation could be demonstrated in patients with RA [28]. These findings theoretically raise the possibility that the local or systematic induction of IDO activity could be tested in inflammatory joint diseases.

## Abbreviations

1-MT = 1-methyl-tryptophan; CFA = complete Freund's adjuvant; CIA = collagen-induced arthritis; CII = type II collagen; CTLA-4 = cytotoxic T lymphocyte-associated antigen-4; ELISA = enzyme-linked immunosorbent assay; IDO = indoleamine 2,3-dioxygenase; IFN-γ = interferon-γ; IgGAM = immunoglobulins G, A, and M; IL = interleukin; i.p. = intraperitoneally; K = kynurenine; NK = natural killer; RA = rheumatoid arthritis; T = tryptophan.

## Competing interests

The authors declare that they have no competing interests.

## Authors' contributions

SS performed the experimental work and prepared the manuscript. TK performed the experimental work. ZS advised on the study. KM and TTG are the heads of laboratory, supervised the experimental work, and advised on the study. JV is the senior researcher and supervisor of the experimental work and advised on the study. All authors read and approved the final manuscript.
